# Identification of the calcitonin receptor in osteoarthritic chondrocytes

**DOI:** 10.1186/1756-0500-4-407

**Published:** 2011-10-13

**Authors:** Toni Segovia-Silvestre, Caroline Bonnefond, Bodil C Sondergaard, Tjorbjoern Christensen, Morten A Karsdal, Anne C Bay-Jensen

**Affiliations:** 1 Nordic Bioscience A/S, Herlev Hovedgade 207, 2730 Herlev, Denmark; 2 Gentofte University Hospital Orthopedic Surgery Unit, 2820 Gentofte, Denmark

## Abstract

**Background:**

Preclinical and clinical studies have shown that salmon calcitonin has cartilage protective effects in joint degenerative diseases, such as osteoarthritis (OA). However, the presence of the calcitonin receptor (CTR) in articular cartilage chondrocytes is yet to be identified. In this study, we sought to further investigate the expression of the CTR in naïve human OA articular chondrocytes to gain further confirmation of the existents of the CTR in articular cartilage.

**Methods:**

Total RNA was purified from primary chondrocytes from articular cartilage biopsies from four OA patients undergoing total knee replacement. High quality cDNA was produced using a dedicated reverse transcription polymerase chain reaction (RT-PCR) protocol. From this a nested PCR assay amplifying the full coding region of the CTR mRNA was completed. Western blotting and immunohistochemistry were used to characterize CTR protein on protein level in chondrocytes.

**Results:**

The full coding transcript of the CTR isoform 2 was identified in all four individuals. DNA sequencing revealed a number of allelic variants of the gene including two potentially novel polymorphisms: a frame shift mutation, +473del, producing a shorter form of the receptor protein, and a single nucleotide polymorphism in the 3' non coding region of the transcript, +1443 C>T. A 53 kDa protein band, consistent with non-glycosylated CTR isoform 2, was detected in chondrocytes with a similar size to that expressed in osteoclasts. Moreover the CTR was identified in the plasma membrane and the chondrocyte lacuna of both primary chondrocytes and OA cartilage section.

**Conclusions:**

Human OA articular cartilage chondrocytes do indeed express the CTR, which makes the articular a pharmacological target of salmon calcitonin. In addition, the results support previous findings suggesting that calcitonin has a direct anabolic effect on articular cartilage.

## Background

*In vitro *studies have shown that salmon calcitonin (sCT) attenuates proteolysis in articular cartilage, inhibiting collagenase and phospholipase A2 activities [1,2]. Simultaneous induction of the intracellular second messenger cyclic adenosine 3'5'-monophosphate (cAMP) and down-regulation of MMP has been observed in bovine articular cartilage explants in response to sCT [3]. Moreover, sCT has been reported to induce anabolic effects on cartilage formation, proteoglycan and glycosaminoglycan syntheses in chondrocytes and growth plate cultures from different species [4-7]. *In vivo*, long-term sCT treatment has been shown to increase the number of hypertrophic chondrocytes and thickness of the epiphyseal plate in young rats [8]. In addition, a number of studies have documented the palliative effects of CT in different joint damage animal models [9-12]. Finally, the effect of sCT on cartilage degradation and pain alleviation was recently demonstrated in different clinical settings where osteoarthritis (OA) patients were treated with salmon calcitonin [13,14]. Thus there are several indications that modulation of the calcitonin receptor (CTR) is a valid target for treatment of joint degenerative diseases. However, the mode of action of which sCT exerts its effect on cartilage need to be further investigated.

The physiological effects of sCT in humans are mediated through high-affinity CT receptors (CTR) - a class B of the G-protein coupled receptor (GPCR) family. The binding of sCT to its receptor produces intracellular accumulation of cAMP [15]. The CTR has been cloned and partially characterized in different cell types and species [16-18], and a number of CTR isoforms have been identified so far. In humans, CTR isoforms are known to arise from alternative splicing of the primary transcript [19,20] of a unique gene (CALCR) located at chromosome 7q21.3 [21]. The two most common human variants differ in a 16-amino acid insert in the putative first intracellular loop [22,23] and this feature confers them different abilities to activate the cAMP and protein kinase C (PKC) signal transduction pathways [24,25]. A different splice variant lacking both the 16-amino acid insert in the first intracellular domain as well as the first 47 amino acids of the amino-terminus extracellular domain was reported to bind to [^125^I] sCT with high affinity and responding to human CT with increases in cAMP [26]. The significance of two most recently discovered isoforms [27] is still unknown. A further human CTR variant results from a T-to-C base mutation producing a leucine 447 to proline (L447P) amino acid change. This substitution has no apparent effect on ligand binding or receptor function *in vitro *[28], but has been associated with decreased fracture risk in postmenopausal women in different studies [29,30]. In summary, CTR variants appear to present different binding properties and ability to couple to signal transduction pathways. Currently, the occurrence, expression regulation and physiological significance of those variants are largely unknown.

The effects of sCT on bone have been demonstrated to be mediated by direct binding of the hormone to CTRs expressed by osteoclasts in their basolateral membrane [31]. In contrast, no conclusive evidence of the expression of the CTR in cartilage has been provided yet. Our group has previously reported the existence of an intron spanning sequence of the coding region of CALCR mRNA in bovine articular cartilage [32]. In contrast, Lin and colleagues concluded that the CTR is not expressed in human cartilage after investigating its existence in chondrocytes cultures by polymerase chain reaction (PCR), western blotting and immunochemical analysis [33]. This controversy has left an important question unanswered: does the effect of sCT on articular cartilage occur via a direct interaction with cartilage cells or through an indirect route via inhibition of resorptive activity of osteoclasts in subchondral bone?

In the present study we attempted to test the hypothesis that the CTR is expressed in human articular cartilage. We used a specific RT-PCR DNA sequencing strategy to retrieve and identify the CTR transcript expressed in chondrocytes. We then sought to assess the expression and identity of a CTR protein consistent with the identified transcripts by western blotting and to determine its subcellular localization by immunochemical analysis.

## Methods

### Patients' characteristics

This investigation originally included 21 OA patients (17 females and 4 males) with an average age of 66 ± 11 years undergoing elective total knee joint replacement surgery at the Orthopedic Surgery Unit of Gentofte hospital (Denmark). Given the characteristics of the study samples and the molecular methods performed, individual samples could not be used for simultaneous genetic and protein analysis. Therefore, results presented in this paper correspond to four patients of the study group in which complete DNA sequencing could be optimally performed. Four other patients were used for western blotting analysis, and two different patients for immunochemical analysis. The study was approved by the Ethics Committee of the Capital Region of Denmark, DK-3400 (approval no. HD-2007-0084). Patients were informed about the purpose of the study and gave written consent.

### Chondrocytes isolation from fresh human cartilage tissue

Articular cartilage from non-eroded areas with a normal smooth surface and hard texture was isolated and used for experiments. Dissected specimens free of bone tissue were collected in DMEM (Lonza, Basel, Switzerland) and preserved at 4°C until shipment to the laboratory within 24 hours following surgery, then washed in Dulbecco's PBS buffer (Lonza) and used immediately for chondrocyte cells isolation. Cartilage was cut in 1 mm^2 ^pieces and sequentially digested with 0.5% pronase (Sigma-Aldrich, Dorset, UK) for one hour and 0.5% collagenase type 2 (Wako, Osaka, Japan) overnight at 37°C. The presence of viable chondrocytes was assessed by microscopic observation. Cell pellets were directly used for RNA isolation, western blotting or immunocytochemical analysis or, alternatively, snap frozen in liquid nitrogen and stored at -70°C for later use.

### RNA extraction from isolated chondrocytes

After careful optimization of the RNA extraction from cartilage procedure, then RNA was isolated from four different patients. Cell lysis and RNA extraction were performed using the High-Pure RNA Isolation kit and reagents (Roche, Basel, Switzerland) following manufacturer's instructions. Six to 25 μg of high quality RNA was obtained from an average of 6 million cells, resuspended in 200 μL of PBS and lysed in 400 μL of lysis/binding buffer during 20 min of incubation on ice and frequent vortexing. RNA was eluted in 50 μL of EB buffer (10 mM Tris-Cl, pH 8.5). RNA integrity, quality and quantity were assessed using an Agilent 2100 Bioanalyzer (Agilent Technologies, Santa Clara, CA, USA).

### Reverse transcription polymerase chain reaction

Complementary DNA (cDNA) copies of chondrocyte RNA were prepared using the Transcriptor First Strand cDNA Synthesis kit (Roche). One microgram samples of high quality RNA (RNA Integrity Number (RIN) > 8, concentration > 50 ng/μL, no strong secondary peaks in the electropherogram, A260/280 > 1.8) were used as template in single cDNA synthesis reactions. Combined random hexamer plus anchored-oligo(dT)_18 _primers were used in a pre-reaction mix containing RNA template and primers were first heat denatured for 10 min at 65°C. Subsequently, a preincubation step at 25°C for 10 minutes was introduced before the reverse transcription reaction was performed at 50°C for 1 hour. RT-PCR negative controls were carried out in parallel by substituting reverse transcriptase with water.

### CTR polymerase chain reaction assay

The NCBI Reference Sequence NM_001742.2 was used as template for CALCR PCR assays design. A nested PCR strategy was adopted to amplify the entire coding region of the CTR with specificity and high reaction yields. Briefly, primers (Eurofins MWG Operon, Ebersberg, Germany) for the primary amplification from 2 μL of cDNA preparation were CALCR_F (5'-CCAGTGACAGAATTCCAGGAC, sense) and CALCR_R (5'-GTCTCCCAAAGCAACAGTACC, antisense). Nested primers CALCR_NF (5'-CCAGGACAAAGAGATCTTCA, sense) and CALCR_NR (5'-CAGGAAATGATGGCTCAGTG, antisense) were used to amplify an internal target from 2 μL of the primary amplification product. Herculase II Fusion DNA polymerase (Stratagene, La Jolla, CA, USA) was used in those long range PCR experiments. PCR amplifications were performed in a DYAD dual block PCR machine (MJ Research, Waltham, MA, USA) and consisted of 35 cycles of: 2 minutes denaturation at 95°C; 20 seconds annealing at 54°C for CALCR_NF/CALCR_NR and at 53°C for CALCR_F/CALCR_R; 49 seconds extension at 72°C for CALCR_NF/CALCR_NR and at 50 seconds for CALCR_F/CALCR_R. Negative controls were matched to all samples amplified. Negative control reactions to detect genomic and pseudogene DNA amplification were performed by omitting reverse transcriptase in RT-PCR reactions. Negative controls for laboratory contamination were performed using water as template in PCR reactions.

### DNA sequencing of nested PCR products

DNA sequencing was performed in samples of patient cDNA amplified by nested PCR. The 1500 bp amplicon generated by the CALCR_NF/CALCR_NR assay was purified from the PCR reaction using the QIAquick PCR purification Kit (Qiagen, Hilden, Germany). Approximately 30-60 ng of purified products were sequenced using Bigdye Terminator chemistry in ABI 3730XL capillary sequencers (Applied Biosystems, Foster City, CA, USA) at Eurofins MWG Operon. Overlapping sequencing primers were designed in the 147-1647 nucleotide region of NM_001742.2 using Eurofins MWG Operon online tool. Primers covered both DNA strands of the nested PCR product aiming at excluding polymerase-mediated DNA sequence changes during PCR. For those samples showing polymorphisms or mutations a second confirmatory DNA sequencing experiment was performed starting from stored cDNA material of the patient.

### Western blotting analysis

Chondrocyte pellets of about 6 million of cells were homogenized in 50 μL of RIPA buffer (140 mM NaCl, 10 mM Tris, pH 8, 1 mM EDTA, 1% Triton X-100, 0.1% SDS, 0.1% deoxycholic acid) containing Complete mini EDTA-free protease inhibitor cocktail (Roche) and incubated for 5 min on ice. Human osteoclasts were differentiated for 12 days from CD14+ monocytes purified from human blood (Rigshospitalet Bloodbank, Copenhagen, Denmark) using Receptor Activator for Nuclear Factor κB Ligand and Macrophage colony-stimulating factor as previously described [34]. Pellets were lysed in 500 μL RIPA buffer. Ten to 20 μg total protein of each lysate preparation was electrophoresed in polyacrylamide gels in denaturating condition (10% dithiothreitol) with Full Range Rainbow protein marker (GE Healthcare, Buckinghamshire, UK). Proteins were blotted on nitrocellulose membranes (Whatman, Kent, UK), soaked in 10 mM N-cyclohexyl-3-aminopropanesulfonic acid (Sigma) plus 10% ethanol pH 11, and incubated for 1 hour in blocking buffer (TBS 0.1% Tween-20 + 5% skimmed milk powder). Nitrocellulose membranes were probed for 2 hours with primary antibodies at different dilutions in blocking buffer. After blocking, membranes were probed for 2 hours with anti-human CTR antibodies from different suppliers: SP1338P (Acris, Herford, Germany) diluted 1:200; H00000799-M01 (Abnova, Taipei City, Taiwan) diluted 1:200, 250618 (Abbiotec, San Diego, CA, USA) diluted 1:200 and Ab11042-50 (Abcam, Cambridge, MA, USA) diluted 1:2000. Subsequently, membranes were incubated for one hour at room temperature with secondary antibody, either rabbit anti-mouse 315035045 or goat anti-rabbit 111035003 (The Jackson Laboratory, Bar Harbor, ME, USA) diluted 1:10000. The Pierce ECL western blotting substrate (Thermo Fisher Scientific, Waltham, MA, USA) was used for detection and activated membranes were revealed with Amersham ECL HyperFilm (GE Healthcare) autoradiography films.

### Immunochemical localization of the human CTR

Primary chondrocytes were isolated from cartilage as described above. The cells were fixed in 4% formalin (Lillie's fluid, Sigma-Aldrich, Glostrup, Denmark) for 10 minutes and rinsed in PBS. Endogenous peroxidases were blocked with 1% hydrogen peroxide in PBS for 20 minutes, followed by rinsing in TBS (Tris buffer saline). Next the cells were incubated with a rabbit polyclonal antibody raised against CTR (SP1338P, Acris antibodies, Herford, Germany), diluted 1:500 and incubated for 1 hour, including blocking agent (BSA, Sigma-Aldrich, Roedovre, Denmark). After rinsing in TBS, 5 drops of peroxidase-labeled EnVision+ anti-rabbit (Dako, Roedovre, Denmark) was added and incubated for 30 minutes. The cells were then rinsed and Dako DAB+ chromogen was add and the color reaction was stopped after 5 minutes, by rinsing in milli-Q water. The cells were counterstained in Mayer's Hematoxylin for 1 minute, rinsed in tap water and ethanol. The stained cells were smeared on to a slide and covered with cover glass. Before isolation of the primary cells from cartilage, small pieces of the tissue were separated and fixed overnight in 4% formalin. The tissues were then embedded in paraffin and cut in 5 micrometer sections. They were then deparaffinated, endogenous peroxidase activity was blocked in 0.4% H_2_O_2 _in 99% ethanol and samples were rehydrated and unmasked for 2 hours in citrate buffer, pH 6 at 60°C. The sections were then incubated overnight at 4°C with polyclonal antibody diluted 1:100 in 1% casein blocking agent (Sigma-Aldrich, Roedovre, Denmark). Sections were then rinsed and peroxidase-labeled EnVision+ anti-rabbit (Dako, Roedovre, Denmark) was added and incubated for 30 minutes. The sections were then rinsed, Dako DAB+ chromogen was added and the color reaction was stopped after 10 minutes by rinsing in Milli-Q water. Finally, sections were counterstained in Mayer's Hematoxylin for 20 seconds, rinsed in tap water, dehydrated, mounted in Kaiser's glycerin jelly and covered. Both primary cells and cartilage sections were assessed under the microscope using CellA v2.7 software (Olympus, Ballerup, Denmark).

## Results

### Human chondrocytes express isoform 2 of the CTR

**Figure 1:**
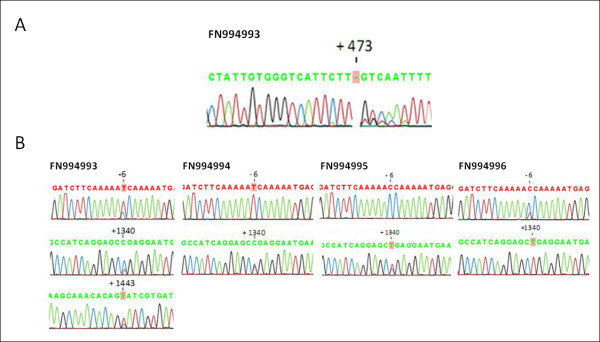
**Allelic variants of CTR isoform 2 found in human chondrocytes**. (A) A novel CALCR frameshift mutation was found in an OA patient. Electropherogram showing a heterozygous single nucleotide deletion at position +473 in CALCR cDNA amplified from primary chondrocytes. Note the appearance of a +1 frameshift in one of the alleles after nt +473. (B) Electropherograms showing the presence of CALCR single nucleotide polymorphisms in four different patients. Three C>T transitions were found at previously reported positions nt -6 (5'-UTR region) and +1340 (coding sequence), and unreported position nt +1443 (3'-UTR region). The complete sequences have been deposited at the INSDC nucleotide database (accession numbers FN994993 to FN994996).

Chondrocytes isolated by enzymatic digestion were used to obtain high quality cartilage RNA for RT-PCR analysis, which was converted to cDNA. The nested PCR reaction (1:CALCR_F/CALCR_R; 2:CALCR_NF/CALCR_NR) produced nanogram amounts of a single 1.5 kbp amplicon. Purification and sequencing of this band confirmed the presence of the full coding sequence of the CALCR isoform 2 in the four patients included. The sequences have been annotated and deposited at the INSDC nucleotide database (accession numbers FN994993 to FN994996). A number of signature polymorphisms were observed in sequencing electropherograms of those transcripts. Remarkably, FN994993 presented a heterozygous nonsense mutation at nt +473, upstream of the start codon (Figure 1A). A traceable n+1 secondary reading frame, denoting the presence of two distinct mRNA species, can be seen starting from the deleted T nucleotide. Three different C-to-T single nucleotide polymorphisms (SNP) were also found at positions -6, +1340, +1443 (Figure 1B).

### Chondrocytes synthesize a 53 kDa CTR protein consistent with isoform 2 of the CTR

**Figure 2:**
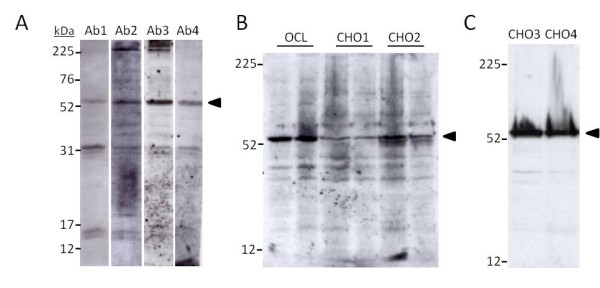
**Western blot detection of CTR in human chondrocytes**. (A) Screening of a panel of anti-CTR antibodies against human osteoclast lysates. A 53 kDa band (arrow), consistent with the receptor's molecular weight, was chosen to evaluate antibody performance. Ab1: H00000799-M01 diluted 1:200; Ab2: SP1338P dil. 1:200; Ab3: 250618 dil. 1:200; Ab4: Ab11042-50 dil. 1:2000. (B) Simultaneous detection of the 53 kDa CTR band in human osteoclasts and chondrocytes OCL: 10 μg and 20 μg protein of *in vitro *differentiated human osteoclasts lysates [34]; CHO1: 30 μg and 15 μg protein of chondrocytes lysates from the same individual; CHO2: 30 μg and 15 μg protein of chondrocytes lysates from a second individual. Antibody: Ab3, dil. 1:200. (C) Detection of CTR in 20 μg protein cell lysates samples of freshly isolated chondrocyte from two different individuals. Antibody: Ab4, dil. 1:2000.

A panel of anti-CTR antibodies from different providers was tested in osteoclasts lysates to identify consensus immunoreactive band(s) attributable to the CTR. Four antibodies detected a 53 kDa band consistent with the molecular weight of the osteoclast CTR (Figure 2A). Western blots of parallel samples of chondrocytes and osteoclasts lysates retrieved the same 53 kDa band in both cell types. CTR levels in chondrocytes were lower for the same amount of total protein loaded (Figure 2B). Subsequent analysis of fresh chondrocyte preparations demonstrated the presence of sizeable amounts of CTR protein in those cells (Figure 2C).

### The CTR protein is localized the plasma membrane of primary chondrocytes

**Figure 3:**
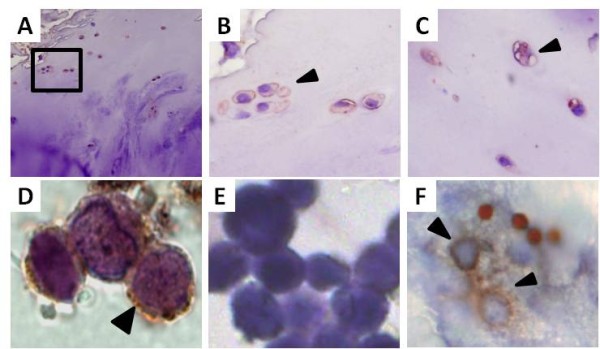
**Immuno-localization of the CTR in human chondrocytes**. (A) Human OA cartilage tissue, upper zone *(obj. 20×)*. The square indicated the area of interest of the upper zone. (B) Clonal chondrocytes (clusters) positive for the CTR *(obj. 40×)*. (C) Singular chondrocytes of the upper zone positive for the CTR *(obj. 40×)*. (D) Overview picture of the deep zone with the tidemark marked in light pink *(obj. 20×)*. (E) Close up of a column chondrocytes of the deep zone *(obj. 100×)*. (F) Close up of a hypertrophic chondrocytes in the deep zone with positive staining in the fragmentized nucleus *(obj. 100×)*. (G)-(I) Positive CTR staining in the cell membrane of primary chondrocytes of three different patients *(obj. 100×)*. (J) Negative control: No staining is shown when omitting the primary antibody in the experimental protocol *(obj. 100×)*. (K) Positive control: A differentiated osteoclast resorbing on bone shows the expected staining of the basolateral membrane where the receptor is expressed *(obj. 100×)*. (L) Negative staining in synovial membrane *(obj. 20×)*. Antibody staining: Acris sp1338p (red-brown) and positive staining is indicated with arrows. Cell nucleus was counter stained with Mayer's hematoxylin (blue).

The expression of the CTR was further investigated by immunohistochemistry. Immunoreactivity was observed in the plasma membrane of proliferative chondrocytes close to the articular surface (Figure 3A, B and 3C). The staining was seen as a dark brown ring following the cellular membrane. Similar staining was observed for primary chondrocytes, although the signal was also observed in the cytosol (Figure 3D). The cytosol staining was probably due the fact that the cell membrane has been permeabilized when isolated from the extracellular matrix by the detergents added. In many instances the cell surface was ruffled and due to the harsh handling of the tissue. Staining using isotype antibody control was negative (Figure 3E). Osteoclasts, isolated with magnetic beads (seen as round spots) and grown on bones slices, were used as positive controls (Figure 3F). Immunoreactivity was observed intracellularly in this multinuclei cell. The osteoclast was seated in a resorption pit; thus the surrounding bone becomes unfocused. No-antibody control was negative (data not shown).

## Discussion

The results presented here are the strongest evidences published to date of the expression of calcitonin receptors in human articular cartilage. The nested PCR assay developed in this study led to the sequencing of the full coding sequence of CALCR gene transcripts of cartilage of OA patients. The CTR isoform 2 was consistently found in all four patients examined. By aligning experimental data to the NCBI Reference Sequence NM_001742.2, a number of genetic features became apparent. We confirmed the presence of two frequent polymorphisms at positions -6 C>T and +1340 C>T. The former has been previously reported but not associated with any particular phenotype [35], while the later provokes a change in amino acid in the CTR protein, L447P that has been linked to bone mineral density (BMD) and osteoporosis (OP). In particular, heterozygosity at L447P has been linked to higher BMD at the femoral neck [36].

Interestingly, three patients in our study were heterozygous for L447P. Restriction fragment length polymorphism analysis of Japanese patients previouly identified this CALCR polymorphism as arising from a single nucleotide substitution leading to either a proline (CC phenotype), leucine (TT) or heterozygote (TC) genotype at amino acid position 447 [37]. Similar studies have been performed in different population such as postmenopausal Italian [38], Caucasian [39] and Polish [40] women, as well as in Caucasians, African-Americans, Asians and Hispanics [41] in order to assess the prevalence of those genotypes and investigate their potential association to BMD. While their authors extract different conclusions, these studies taken together suggest that the human CALCR gene plays a role in BMD and, ultimately, in the incidence of OP.

A novel polymorphism was found at nt +1443 C>T in the 3' untranslated region of the CALCR mRNA. The region contains regulatory sequences targeted by transcription factors, but the effect of such nucleotide change in the expression of the transcript is unknown. The most dramatic finding in the transcript sequences analyzed was a heterozygous missense mutation at position nt +473 caused by the deletion of a T base. This mutation provokes a frame shift in protein translation at amino acid residue 158 leading to early termination at residue 162, located in the first trans-membrane region of the receptor. It would be of interest to study the effect of such mutation and determine its cellular phenotype. Incidentally, a recent genetic association study concluded that a CA dinucleotide polymorphism in the calcitonin gene (CALCA) is associated with risk of developing knee OA, reinforcing the idea that the CT system is deeply involved in cartilage health [42].

In an initial screening we found a common band of approximately 53 kDa in osteoclasts lysates detected by different antibodies. This same band was also retrieved in chondrocytes. The theoretical molecular weight of the mature protein produced by isoform 2, based on its amino acid sequence, is 55 kDa. Four putative *N*-glycosylation sites, i.e. N28, N73, N125 and N130 are present in the amino terminal extracellular domain of the human CTR (UniProtKB/Swiss-Prot P30988). The western blot presented here, performed on freshly isolated chondrocyte pellets, showed a high molecular weight material smear above the 53 kDa bands, but most of the immunoreactivity appears as a single discrete band (Figure 2C). This would suggest that those glycosylation sites are not modified neither in human chondrocytes nor osteoclasts, in contrast to previous findings in heterologous expression studies of human CTR in monkey kidney COS-1 cells [43]. This same blot shows that chondrocytes are indeed capable of strong expression of CTR protein. Paradoxically, this strong expression was demonstrated using the same antibody used by Lin and coworkers [44]. The definitive confirmation of the expression of the CTR protein in human chondrocytes could be obtained by combined immunoprecipitation and mass spectrometry, but it was out of the scope of this study. These results were supported by the localization of the receptor to articular chondrocytes in cartilage sections from OA patients. Next it would be interesting to investigate to what degree different chondrocyte subpopulations express different amounts of CTR and whether the CTR is associated with other phenotype-specific proteins.

The results presented here allow us to conclude that human cartilage does express the calcitonin receptor. The main reason why other studies failed to find similar results may be the cell phenotype used in those investigations. The study published by Lin and coworkers was performed using cultured chondrocytes [45]. Other authors have provided evidences that culturing chondrocytes as an adherent monolayer invariably leads to a process of dedifferentiation whereby cells adopt a fibroblastic morphology, lose their chondrocyte-specific gene expression pattern and initiate or upregulate the expression of fibroblast-associated genes such as type I, III and V collagens and versican [46-48]. Therefore, chondrocytes dedifferentiated on plastic with fetal bovine serum is not recommendable as a representative system for cartilage cell biology studies, particularly in regards to gene expression profile.

## Conclusions

As mentioned above, there are clear evidences of the beneficial effects of calcitonin in OA. However, its mode of action has not been yet fully clarified. OA is a complex disease that affects both bone and cartilage. Our results suggest that, beyond an indirect effect on cartilage via subchondral bone turnover [49], calcitonin may directly target chondrocytes in articular cartilage. Human cartilage cells do express the CTR. This will be confirmed in future clinical studies including a larger number of patients. The identification of isoform 2 in OA human cartilage allows for more targeted pharmacological investigations into the mode of action of calcitonin in OA treatment and prevention. Further research is also warranted into the intracellular mechanisms by which calcitonin protects chondrocytes, increases extracellular matrix synthesis and inhibits cartilage degradation.

## List of abbreviations used

BMD: bone mineral density; CALCA: calcitonin-related polypeptide alpha gene; CALCR: calcitonin receptor gene; cAMP: cyclic adenosine 3'5'-monophosphate; cDNA: complementary DNA; CT: calcitonin; CTR: calcitonin receptor protein; DMEM: Dulbecco's modified Eagle's medium; EB: elution buffer; EDTA: ethylenediaminetetraacetic acid; FCS: fetal calf serum; GPCR: G protein-coupled receptor; MMP: matrix metalloproteinase; OA: osteoarthritis; OP: osteoporosis; PBS: phosphate buffered saline; PCR: polymerase chain reaction; PKC: protein kinase C; RIN: RNA integrity number; RIPA: radioimmunoprecipitation assay; RT-PCR: reverse transcription polymerase chain reaction; sCT: salmon calcitonin; SDS: sodium dodecyl sulfate; SNP: single nucleotide polymorphism; TBS: Tris buffer saline; UTR: untranslated region.

## Competing interests

Morten Asser Karsdal owns stock in Nordic Bioscience. Anne C. Bay-Jensen and Bodil C. Sondergaard are full-time employees at Nordic Bioscience. Toni Segovia-Silvestre and Caroline Bonnefond were full-time employees at Nordic Bioscience at the time the work was performed, but are unaffiliated with Nordic Bioscience at the time of submission. Nordic Bioscience is involved in the development of oral salmon calcitonin for the treatment of OP and OA. Caroline Bonnefond and Tjorbjoern Christensen declare that they have no competing interests.

## Authors' contributions

TSS directed the study and drafted the first version of the manuscript. MAK had the original idea for the manuscript and participated in discussing and drafting the manuscript. CB performed genetic and western blot experiments and participated in drafting the last version of the manuscript. ACBJ and BCS performed immunochemical analyses and drafted the final version of the manuscript. TC performed surgical procedures, characterization of patients and participated in drafting the final version of the manuscript. All authors read and approved the last version of the manuscript.
